# Analysis of prognostic factors of metastatic endometrial cancer based on surveillance, epidemiology, and end results database

**DOI:** 10.3389/fsurg.2022.1001791

**Published:** 2023-01-06

**Authors:** Meng Zhang, Ruiping Li, Shan Zhang, Xin Xu, Lixin Liao, Yan Yang, Yuzhen Guo

**Affiliations:** Department of Gynecology, Second Hospital of Lanzhou University, Lanzhou, China

**Keywords:** endometrial cancer, metastasis, SEER database, nomogram, predictive model fund program: natural science foundation of gansu province(17JR5RA242)

## Abstract

**Objective:**

To explore the risk factors for survival and prognosis of patients with metastatic endometrial cancer and to build and verify a reliable prediction model.

**Methods:**

We retrospectively analyzed patients diagnosed with metastatic endometrial cancer in the US Surveillance, Epidemiology, and End Results (SEER) database between January 2010 and December 2015. Univariate and multivariate Cox regression analyses were used to assess clinical variables impact on survival and to construct nomograms. The results of the consistency index (C-index), subject operating characteristic (ROC) curve, and calibration curve were used to evaluate the predictive ability of the nomogram.

**Results:**

This study included 3,878 patients with metastatic endometrial cancer. In the univariate analysis, variables associated with overall survival (OS) and cancer-specific survival (CSS) included age, race, marital status, pathological type, pathological grade, T-stage, N-stage, surgery, radiotherapy, chemotherapy, bone metastasis, brain metastasis, liver metastasis, and lung metastasis. In the multivariate analysis, age, race, pathological type, pathological grade, T-stage, N-stage, surgery, radiotherapy, chemotherapy, brain metastasis, liver metastasis, and lung metastasis were independent risk factors for OS and CSS (all *P *< 0.05). Combined with the results of the multiple factors, the 1-, 3-, 5-, and 8-year nomograms were constructed. For OS and CSS, T-stage had the greatest impact on the adverse prognosis of patients with metastatic endometrial cancer. The C-indexes of the OS and CSS nomograms in the training cohort were 0.749 (95% CI, 0.739–0.760) and 0.746 (95% CI, 0.736–0.756), respectively. The C-indices of OS and CSS in the validation cohort were 0.730 (95% CI, 0.714–0.746) and 0.728 (95% CI, 0.712–0.744), respectively. The ROC curve revealed our model's good prediction accuracy and clinical practicability. The calibration curve also confirmed the consistency between the model and actual existence. The Kaplan-Meier curves revealed statistically significant differences between the risk subgroups (*P *< 0.05).

**Conclusion:**

Our SEER-based nomograms for predicting survival in patients with metastatic endometrial cancer were helpful for the clinical evaluation of patient prognosis.

## Introduction

Endometrial cancer is one of the three major malignancies of the female reproductive system and is most common in perimenopausal and postmenopausal women. In recent years, the incidence of endometrial cancer has been increasing annually, and it increases gradually with age. According to some studies, the risk of illness at 40–59 years old is 0.77%, that at 60–69 years old is 0.87%, and that at 70 years is 1.24%. Elderly patients are often diagnosed at a late stage with poor histological characteristics (1). Multiple risk factors are associated with the development of endometrial cancer, including gene mutation, chronic estrogen stimulation, and lifestyle changes (such as obesity, diabetes, and hypertension), among which obesity is an important independent risk factor for endometrial cancer. According to statistics, nearly half of the patients with endometrial cancer are associated with obesity (2). One study showed that the relative risk of endometrial cancer in women with metabolic syndrome is 1.89 (3). The traditional “binary” classification divides endometrial cancer into types I and II. However, in patients with high-risk endometrial cancer, the 5-year survival rate drops to 10%–20% (4).

Surgery is the gold-standard treatment for endometrial cancer (5). The scope of surgery was determined using preoperative risk assessment (6). Among these, the standard treatment methods limited to the uterus are total hysterectomy and bilateral tubal oophorectomy, and adjuvant treatment is used for patients with high-risk factors. Approximately 20% of high-risk patients have lymph node metastasis. Lymph node status is closely related to prognosis. Radical primary tumor resection combined with extensive lymph node dissection remains the standard of care for most tumors (7). Current European guidelines recommend pelvic lymph node dissection and infrarenal para-aortic lymph node dissection for high-risk endometrial cancer (8). Some studies have suggested that systematic lymphadenectomy cannot improve patient prognoses and may increase the risk of intraoperative and postoperative complications (including lymphocytosis, lymphedema, and hemorrhage), especially in elderly and obese patients (9, 10). Controversies remain regarding the indications, resection scope, and therapeutic value of lymphadenectomy in disease management (11). Sentinel lymph node localization technology can improve the detection rate of lymph node metastasis, reduce the false negative rate, and, to some extent, positively impact the prognosis of high-risk endometrial cancer patients (8). Recently, many studies have demonstrated the importance of sentinel lymph node detection in various malignant tumors, which may significantly improve patients' survival rate and quality of life (12).

The Surveillance, Epidemiology, and End Results (SEER) database is an open database established by the National Cancer Institute of the United States, covering approximately 34.6% of the population in the United States. It collects information about the incidence, treatment, prognosis, and mortality of cancer patients, and provides important clinical guidance. In medical research and clinical practice, nomograms are increasingly used as tools to assess the risk and prognosis of disease occurrence (13). Based on the SEER database, this study retrospectively analyzed the clinical data of 3,878 patients with distant metastasis of high-risk endometrial cancer between January 2010 and December 2015. To study the factors affecting prognosis, construct nomograms and verify them, to provide a theoretical basis for survival and prognostic analysis of patients.

## Materials and methods

### Patients

From the SEER database, we identified all endometrial cancer patients with EC with distant organ metastasis at the first visit between January 2010 and December 2015. The SEER database is a public database, which does not require ethical review and informed consent.

**Figure 1:**
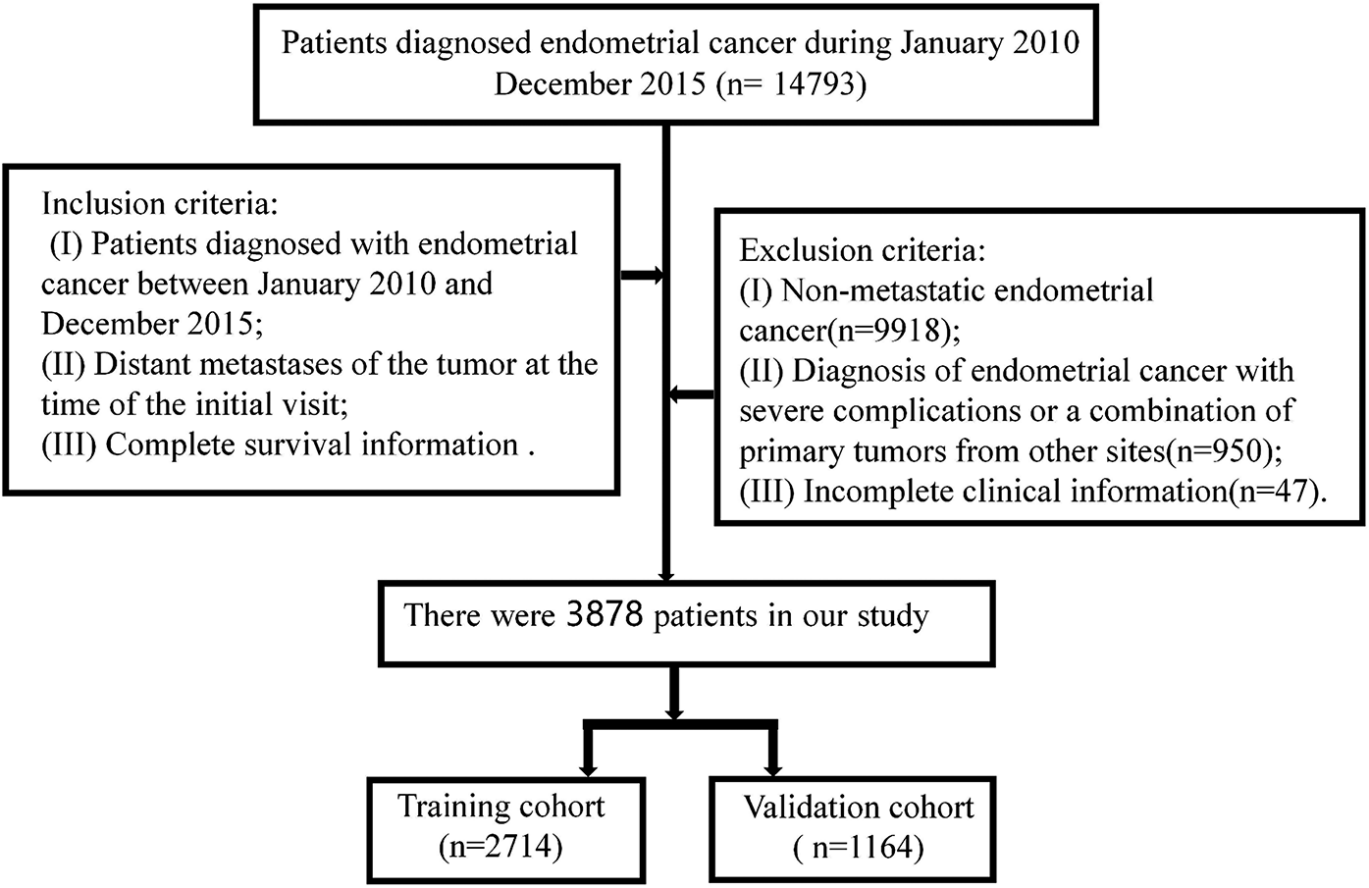
Flow chart for screening patients with metastatic endometrial cancer.

Inclusion criteria: (I) Patients diagnosed with endometrial cancer between January 2010 and December 2015; (II) Patients with distant metastasis at the first visit; (III) Endometrial carcinoma was the only primary tumor; and (IV) Complete survival information. The Exclusion criteria were as follows: (I) Non-metastatic endometrial cancer (*n* = 9918); (II) Diagnosis of endometrial cancer with severe complications or a combination of primary tumors from other sites (*n* = 950); (III) Unknown survival (*n* = 38), survival status (*n* = 6), or surgery (*n* = 3). Patient screening flow chart is shown in Figure 1.

### Study variables

Clinicopathological characteristics collected included patient age, race, marital status, pathological grade, T-stage, N-stage, surgery, radiotherapy, chemotherapy, bone metastasis, brain metastasis, liver metastasis, lung metastasis, type of pathology, overall survival (OS), and cancer-specific survival (CSS). Pathological grades included I (highly differentiated), II (moderately differentiated), III (poorly differentiated), IV (undifferentiated), and unknown, and the seventh edition of the American Joint Committee on Cancer TNM stage was used. The outcome factors included the OS (indicating the total survival) and CSS. For metastatic endometrial cancer, OS was defined as the time from diagnosis to death, and CSS was defined as the time from diagnosis to death due to metastatic endometrial cancer. The initial operation of patients with metastatic endometrial cancer was the starting point of follow-up, and the end point was death or follow-up until December 31, 2018.

### Statistical analyses

Patients eligible for inclusion were randomized into the training cohort (2,714 patients) and the validation cohort (1,164 patients) based on a 7:3 ratio. A *χ*^2^ test was performed to determine the underlying clinicopathological characteristics of the two cohorts. All statistical analyses were carried out using SPSS 24.0 (SPSS Inc., Chicago, IL, United States), and R (R Foundation for Statistical Computing, Vienna, Austria). Analysis using x-tile software showed the optimal age value truncation values. Cox regression analysis was used to determine the prognostic factors for OS and CSS in the training cohort, and independent risk factors were used to construct nomograms of OS and CSS, respectively. The ability of the prediction model was evaluated using the consistency index (C-index) and area under the subject operating characteristic curve (ROC) (AUC). Among them, a C-index of 0.5 indicates that the model had no prediction ability, and 0.5–1.0 indicates that the prediction ability was gradually enhanced. A calibration curve was drawn to evaluate the consistency between the nomogram and actual models. Statistical significance was set at *P* < 0.05.

## Result

### Patient characteristics

Between January 2010 and December 2015, 14,793 patients were diagnosed as endometrial cancer in the SEER database. A total of 3,878 patients were enrolled in this study and randomly divided into a training cohort (2,714 cases) and a validation cohort (1,164 cases) at a ratio of 7:3. The specific clinical data of the included subjects were shown in Table 1.

**Table 1:** Distribution of basic clinical features of patients with metastatic endometrial cancer.

Variables	The training cohort	The validation cohort	Total	Cardinality	*P*
	No. (%)	No. (%)	No. (%)	No. (%)	
Age				0.241	0.623
≤65	1,497 (55.2)	652 (56.0)	2,149 (55.4)		
>65	1,217 (44.8)	512 (44.0)	1,729 (44.6)		
Race				3.925	0.140
White	1,956 (72.1)	803 (69.0)	2,759 (71.1)		
Black	498 (18.3)	233 (20.0)	731 (18.8)		
Other/ Unknown	260 (9.6)	128 (11.0)	388 (10.0)		
Marital status				1.861	0.394
Married	1,158 (42.7)	475 (40.8)	1,633 (42.1)		
Unmarried	619 (22.8)	287 (24.7)	906 (23.4)		
Other/Unknown	937 (34.5)	402 (34.5)	1,399 (34.5)		
Pathological grade				6.152	0.188
I	113 (4.2)	34 (2.9)	147 (3.8)		
II	255 (9.4)	117 (10.1)	372 (9.6)		
III	980 (36.1)	450 (38.7)	1,430 (36.9)		
IV	556 (20.5)	238 (20.4)	794 (20.5)		
Unknown	810 (29.8)	325 (27.9)	1,135 (29.3)		
T stage				5.934	0.313
T0	13 (0.5)	4 (0.3)	17 (0.4)		
T1	398 (14.7)	159 (13.7)	557 (14.4)		
T2	189 (7.0)	63 (5.4)	252 (6.5)		
T3	1,284 (47.3)	580 (49.8)	1,864 (48.1)		
T4	410 (15.1)	166 (14.3)	576 (14.9)		
Unknown	420 (15.5)	192 (16.5)	612 (15.8)		
N stage				0.343	0.952
N0	1,276 (47.0)	546 (46.9)	1,822 (47.0)		
N1	605 (22.3)	256 (22.0)	861 (22.2)		
N2	485 (17.9)	205 (17.6)	690 (17.8)		
Unknown	348 (12.8)	157 (13.5)	505 (13.0)		
Surgery				0.058	0.809
No	952 (35.1)	413 (35.5)	1,365 (35.2)		
Yes	1,762 (64.9)	751 (64.5)	2,513 (64.8)		
Radiotherapy				1.680	0.195
Yes	613 (22.6)	241 (20.7)	854 (22.0)		
No/Unknown	2,101 (77.4)	923 (79.3)	3,024 (78.0)		
Chemotherapy				0.101	0.751
Yes	1,791 (66.0)	762 (65.5)	2,553 (65.8)		
No/Unknown	923 (34.0)	402 (34.5)	1,325 (34.2)		
Bone					
Yes	285 (10.5)	124 (10.7)	409 (10.5)	0.020	0.888
No/Unknown	2,429 (89.5)	1,040 (89.3)	3,469 (89.5)		
Brain				0.115	0.735
Yes	83 (3.1)	38 (3.3)	121 (3.1)		
No/Unknown	2,631 (96.9)	1,126 (96.7)	3,757 (96.9)		
Liver				1.933	0.164
Yes	389 (14.3)	187 (16.1)	576 (14.9)		
No/Unknown	2,325 (85.7)	977 (83.9)	3,302 (85.1)		
Lung				2.986	0.084
Yes	821 (30.3)	844 (27.5)	1,141 (29.4)		
No/Unknown	1,893 (69.7)	320 (72.5)	2,737 (70.6)		
Histology					
Adenocarcinoma	800 (29.5)	347 (29.8)	1,147 (29.6)	0.044	0.834
Other	1,914 (70.5)	817 (70.2)	2,731 (70.4)		

Univariate analysis revealed that age, marital status, race, pathological grade, T-stage, N-stage, surgery, radiotherapy, chemotherapy, bone metastasis, brain metastasis, liver metastasis, lung metastasis, and pathological type were all significant risk factors for OS and CSS. These risk factors were included in the multivariate analysis (*P *< 0.05). Age, race, pathological grade, T-stage, N-stage, surgery, radiotherapy, chemotherapy, brain metastasis, liver metastasis, lung metastasis, and pathological type were independent risk factors for adverse OS and CSS (Tables 2, 3).

**Table 2:** Univariate and multivariate analyses of variables associated with OS in the training cohort (*n* = 2714).

Variables	Univariate analysis			Multivariate analysis		
	HR	95%Cl	*P*	HR	95%Cl	*P*
Age			<0.001			
≤65	Ref			Ref		
>65	1.349	1.242–1.465	<0.001	1.195	1.094–1.304	<0.001
Race			<0.001			
White	Ref			Ref		
Black	1.197	1.034–1.386	0.016	1.148	1.030–1.279	0.012
Other/ Unknown	1.585	1.341–1.872	<0.001	0.912	0.786–1.058	0.223
Marital status			<0.001			
Married	Ref			Ref		
Unmarried	1.051	0.944–1.171	0.364	1.057	0.946–1.181	0.330
Other/Unknown	1.217	1.108–1.337	<0.001	1.030	0.935–1.136	0.548
Pathological grade			<0.001			
I	Ref			Ref		
II	1.124	0.852–1.482	0.408	1.403	1.062–1.854	0.017
III	1.970	1.543–2.514	<0.001	2.170	1.689–2.788	<0.001
IV	1.972	1.534–2.536	<0.001	2.109	1.622–2.741	<0.001
Unknown	2.204	1.724–2.820	<0.001	1.888	1.468–2.427	<0.001
T stage			<0.001			
T0	Ref			Ref		
T1	2.090	0.931–4.692	0.074	2.348	1.042–5.288	0.039
T2	2.692	1.190–6.089	0.017	2.938	1.294–6.668	0.010
T3	2.681	1.202–5.982	0.016	3.521	1.571–7.890	0.002
T4	3.204	1.429–7.181	<0.001	3.540	1.573–7.966	0.002
Unknown	5.667	2.529–12.697	<0.001	3.123	1.389–7.026	0.006
N stage			<0.001			
N0	Ref			Ref		
N1	1.322		<0.001	1.205	1.080–1.344	0.001
N2	1.311		<0.001	1.247	1.109–1.401	<0.001
Unknown	1.738		<0.001	1.055	0.920–1.209	0.446
Surgery			<0.001			
No	Ref			Ref		
Yes	0.342	0.313–0.373	<0.001	0.394	0.354–0.438	<0.001
Radiotherapy			<0.001			
Yes	Ref			Ref		
No/Unknown	1.343	1.213–1.487	<0.001	1.339	1.202–1.492	<0.001
Chemotherapy			<0.001			
Yes	Ref			Ref		
No/Unknown	2.469	2.264–2.264	<0.001	2.254	2.057–2.470	<0.001
Bone			<0.001			
Yes	Ref			Ref		
No/Unknown	0.616	0.541–0.702	<0.001	0.923	0.803–1.061	0.262
Brain			<0.001			
Yes	Ref			Ref		
No/Unknown	0.505	0.401–0.635	<0.001	0.454	0.357–0.577	<0.001
Liver			<0.001			
Yes	Ref			Ref		
No/Unknown	0.562	0.502–0.629	<0.001	0.696	0.619–0.781	<0.001
Lung			<0.001			
Yes	Ref			Ref		
No/Unknown	0.654	0.598–0.715	<0.001	0.829	0.755–0.911	<0.001
Histology			<0.001			
Adenocarcinoma	Ref			Ref		
Other	1.509	1.374–1.658	<0.001	1.323	1.195–1.465	<0.001

**Table 3:** Univariate and multivariate analyses of variables associated with OS in the training cohort (*n* = 2714).

Variables	Univariate analysis			Multivariate analysis		
	HR	95%Cl	*P*	HR	95%Cl	*P*
Age			<0.001			
≤65	Ref			Ref		
>65	0.764	0.702–0.832	<0.001	1.168	1.067–1.279	0.001
Race			<0.001			
White	Ref			Ref		
Black	1.171	1.008–1.359	0.039	1.124	1.005–1.257	0.040
Other/ Unknown	1.523	1.284–1.806	<0.001	0.927	0.796–1.078	0.325
Marital status			<0.001			
Married	Ref			Ref		
Unmarried	0.841	0.763–0.926	<0.001	1.063	0.949–1.192	0.290
Other/Unknown	0.892	0.795–1.000	0.50	1.015	0.918–1.122	0.770
Pathological grade			<0.001			
I	Ref			Ref		
II	0.426	0.328–0.553	<0.001	1.483	1.106–1.990	0.008
III	0.510	0.430–0.604	<0.001	2.298	1.762–2.997	<0.001
IV	0.895	0.807–0.992	0.035	2.206	1.672–2.910	<0.001
Unknown	0.890	0.789–1.003	0.056	2.003	1.535–2.614	<0.001
T stage			<0.001			
T0	Ref			Ref		
T1	0.189	0.084–0.424	<0.001	2.184	0.969–4.923	0.060
T2	0.371	0.371–0.435	<0.001	2.792	1.229–6.343	0.014
T3	0.403	0.403–0.593	<0.001	3.280	1.463–7.353	0.004
T4	0.425	0.425–0.540	<0.001	3.317	1.473–7.469	0.004
Unknown	0.575	0.496–0.667	<0.001	2.935	1.304–6.606	0.009
N stage			<0.001			
N0	Ref			Ref		
N1	0.595	0.522–0.679	<0.001	1.222	1.093–1.367	<0.001
N2	0.798	0.691–0.921	0.002	1.251	1.110–1.411	<0.001
Unknown	0.783	0.673–0.910	0.001	1.021	0.886–1.177	0.776
Surgery			<0.001			
No	Ref			Ref		
Yes	2.883	2.635–3.154	<0.001	0.396	0.355–0.443	<0.001
Radiotherapy			<0.001			
Yes	Ref			Ref		
No/Unknown	0.739	0.665–0.821	<0.001	1.355	1.212–1.515	<0.001
Chemotherapy			<0.001			
Yes	Ref			Ref		
No/Unknown	0.417	0.381–0.456	<0.001	2.208	2.009–2.426	<0.001
Bone			<0.001			
Yes	Ref			Ref		
No/Unknown	1.606	1.403–1.837	<0.001	0.932	0.807–1.076	0.339
Brain			<0.001			
Yes	Ref			Ref		
No/Unknown	1.998	1.578–2.530	<0.001	0.448	0.350–0.573	<0.001
Liver			<0.001			
Yes	Ref			Ref		
No/Unknown	1.788	1.592–2.008	<0.001	0.691	0.613–0.779	<0.001
Lung			<0.001			
Yes	Ref			Ref		
No/Unknown	1.530	1.396–1.676	<0.001	0.824	0.748–0.908	<0.001
Histology			<0.001			
Adenocarcinoma	Ref			Ref		
Other	0.655	0.594–0.721	<0.001	1.346	1.211–1.495	<0.001

### Nomogram development and validation

**Figure 2:**
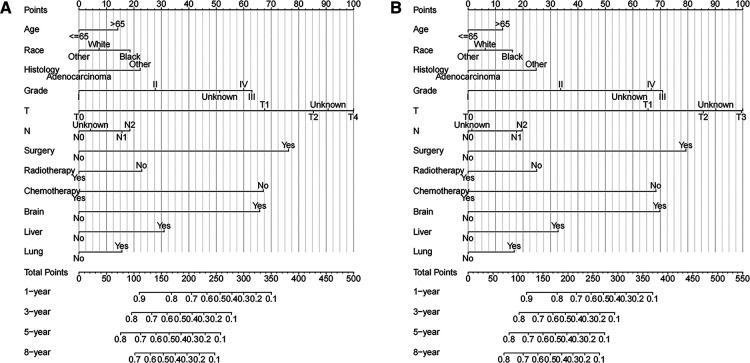
Nomograms of metastatic endometrial cancer to predict 1-,3-,5- and 8-year survival rates (**A**) OS and (**B**) CSS.

**Figure 3:**
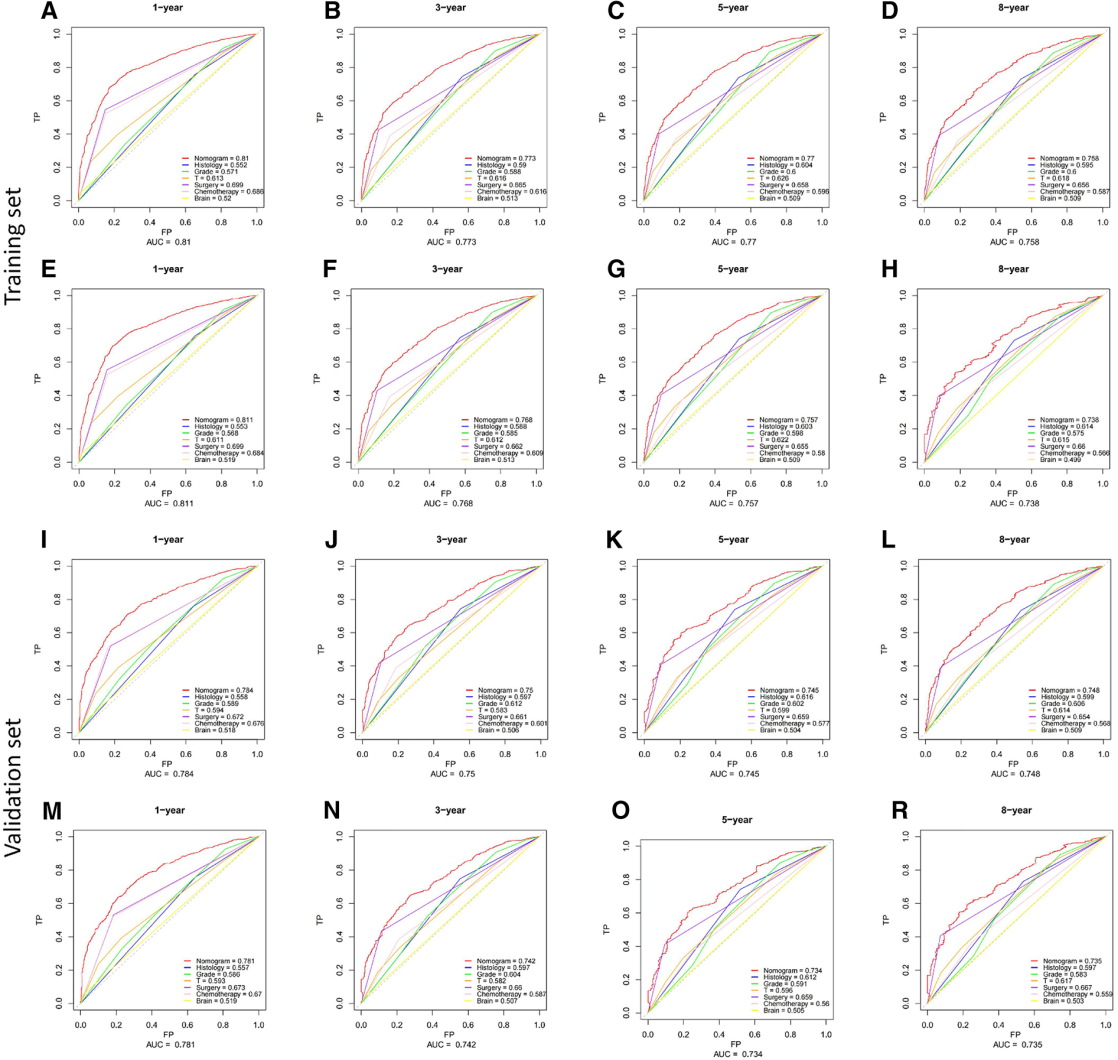
ROC curves of the nomograms for predicting CSS and OS at 1-,3-,5- and 8-year point. (**A–H**) the training cohort; (**I–P**) the validation cohort.

**Figure 4:**
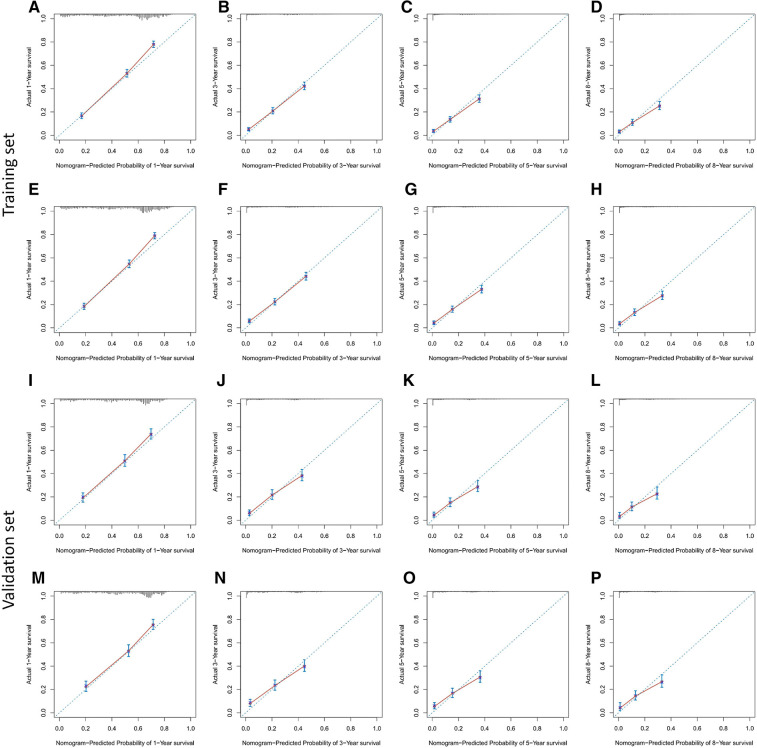
Calibration plots for predicting OS and CSS at 1-,3-,5- and 8-year. (**A–H**) the training cohort; (**I–P**) the validation cohort.

Based on Cox multivariate analysis results, nomograms of the training cohort's OS and CSS were plotted using R. The OS nomogram results showed that pathological grading (grade III) had the greatest impact on the prognosis of patients with metastatic endometrial cancer, followed by surgery (Figure 2A). The CSS nomogram showed that the most influential factor for prognosis was pathological grade (IV), followed by surgery (Figure 2B). We used the C-index, ROC curve, and calibration curve to verify the predictive model. The results of the training cohort showed that the C-index of OS was 0.749 (95% CI, 0.739–0.760), and the C-index of CSS was 0.746 (95% CI, 0.736–0.756); The validation cohort showed that the C-index of OS was 0.730 (95% CI, 0.714–0.746), and the C-index of CSS was 0.728 (95% CI, 0.712–0.744). The results indicated that the prediction model had a high accuracy. The AUC of patients at 1-, 3-, 5- and 8-years were: 0.81, 0.773, 0.77, and 0.758, respectively, for the training cohort (OS) (Figures 3A–D); 0.811, 0.768, 0.757, and 0.738, respectively, for the training cohort (CSS) (Figures 3E–H); 0.784, 0.75, 0.745, and 0.748, respectively, for the validation cohort (OS) (Figures 3I–L); 0.781, 0.742, 0.734, and 0.735, respectively, for the validation cohort (CSS) (Figures 3M–P). In addition, we used calibration curves to further evaluate the accuracy of the prediction model and repeated sampling with bootstrap (B = 1000). The results showed that the calibration curves of the training and verification cohorts (OS and CSS) were close to the 45° diagonal, indicating that the predicted probability was consistent with the actual probability (Figure 4).

### Risk group analysis

**Figure 5:**
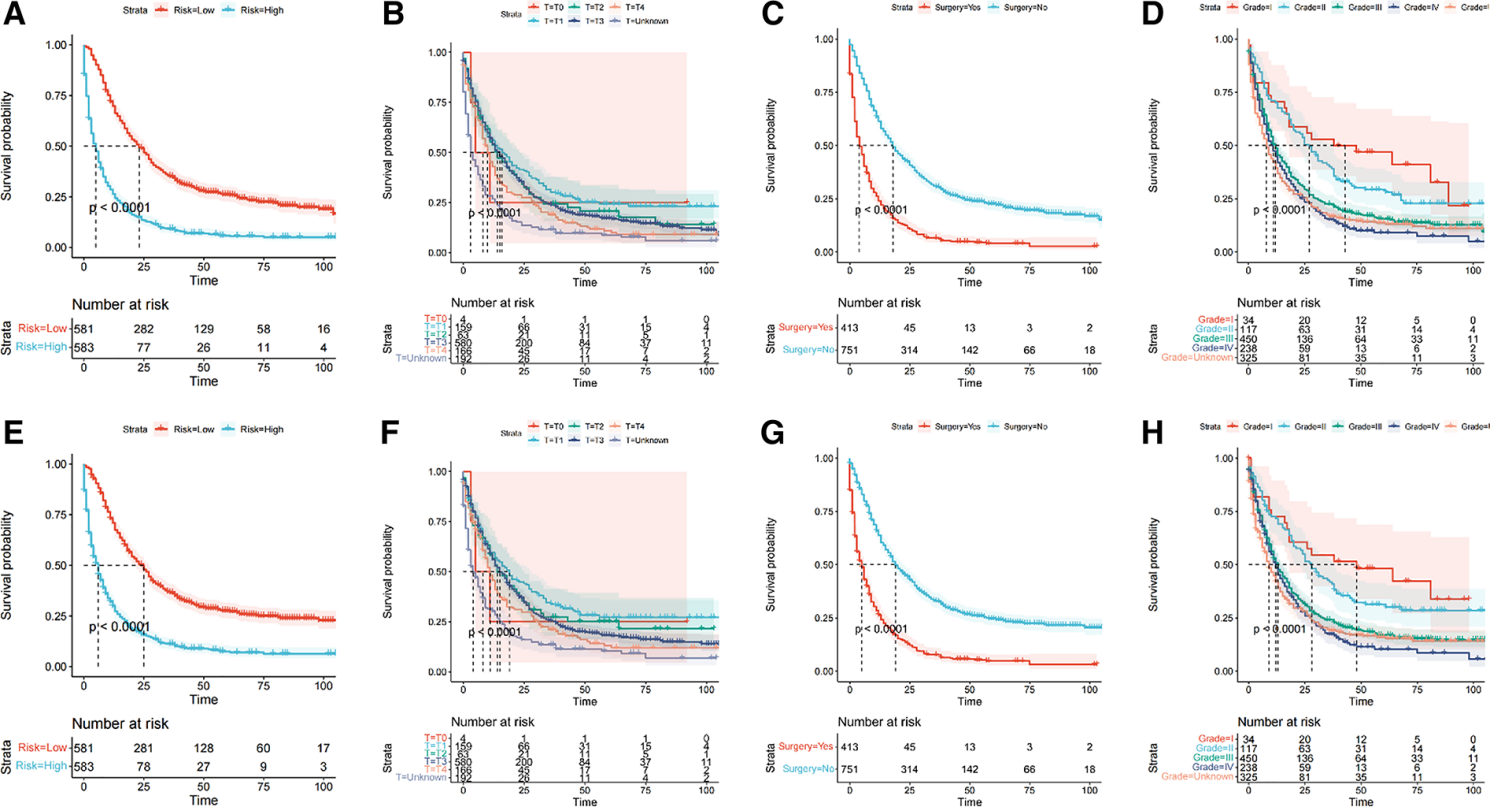
The risk stratification system constructed by Kaplan-Meier survival analysis in the validation cohort. (**A–D**) OS; (**E–H**) CSS.

Kaplan-Meier analysis revealed that T-stage (Figures 5B,F), surgery (Figures 5C,G), and pathological grade (Figures 5D,H) of metastatic endometrial cancer patients were significantly correlated with OS and CSS (*P *< 0.0001). We divided the validation cohort into high- and low-risk groups according to the median total score of the nomograms (OS 169.719, CSS, 172.557). Kaplan-Meier survival analysis showed that the incidence of OS and CSS in low-risk patients was higher than those in high-risk patients (*P *< 0.001) (Figures 5A,E).

## Discussion

With the continuing progress in medical technology, the survival rate of endometrial cancer has significantly improved, and nearly 75% of endometrial cancers can be diagnosed at an early stage. However, in patients with advanced-stage disease, the recurrence rate is relatively high. Recurrence has been detected in approximately 20% of patients with endometrial cancers. The most common recurrence sites are the pelvis and abdomen, and the recurrence time is usually 1–2 years (14, 15). Recurrence remains a major clinical challenge. The purpose of surgical resection of the cancer lesions is to eliminate residual lesions. Combined postoperative radiotherapy and chemotherapy can improve the survival rate of patients. Recent studies have proposed proton beam therapy as a potentially effective method for treating recurrent endometrial cancer (16).

Metastasis is one of the most common causes of death among patients with cancers. Lymph node metastasis is the most common type of metastasis (17). Distant organ metastasis is rare (18). Early and accurate diagnosis is particularly important to establish the best treatment plan and prognosis. Imaging examinations can identify the primary tumor, evaluate the status of lymph node involvement, identify local and distant late local lesions, and detect distant metastatic lesions (19). Positron emission tomography–computed tomography has high sensitivity and specificity for detecting distant metastasis in high-risk patients (100% and 96%, respectively) (20).

Our study showed that in endometrial cancer patients, distant organ metastasis was the commonest in lung metastasis (29.4%), followed by liver metastasis (14.9%), bone metastasis (10.5%), and brain metastasis (3.1%). Brain, liver, and lung metastases were independent prognostic factors for OS and CSS. In single-organ metastasis, lung metastasis had the longest survival time, while brain metastasis had the shortest survival time, which was consistent with previous retrospective study results (21). Bone metastasis was not significant in the multivariate analysis. Hu et al. (22) included endometrial cancer patients with bone metastasis in the SEER database and found that the 1-year OS and CSS rates of these patients with bone metastasis were 33.8% and 35.8%, respectively. Therefore, active management of lung, liver, and brain metastases may help prolong the survival of patients with endometrial cancer. Some studies have proposed that tumor reduction surgery also fails to improve the OS rate of patients (23). In our study, surgery did not improve the survival rate of the patients. Chemotherapy and radiotherapy significantly improve patient survival. This is inconsistent with the results of Hu et al. (22), who found that radiotherapy had no significant impact on the survival of patients with EC in stage IVB. Other researchers have also proposed that brachytherapy can significantly improve the survival of patients with poorly differentiated stage IB endometrial cancer. External radiation radiotherapy and lymphadenectomy can also prolong the survival time of patients, but chemotherapy does not confer survival advantages to high-risk and early endometrial cancer patients (24). Therefore, for patients with metastatic endometrial cancer, formulation of the treatment plan is not simple. With the continuous progress in medical diagnosis and treatment, the survival outcomes of endometrial cancer have significantly improved. Fertility-preserving therapies have become a feasible choice for cancer patients. However, the overall health status of patients receiving surgical treatment is significantly reduced, especially in terms of fertility and sexual function (25). The type of operation, duration of the operation, and postoperative adjuvant treatment have a greater impact on the sexual function of patients. The surgical method of endometrial cancer has changed from traditional open surgery to minimally invasive surgery. Some studies have shown that the sexual function is significantly reduced after laparotomy compared with that after laparoscopic surgery; compared with patients receiving brachytherapy alone, patients receiving brachytherapy and external radiation report significantly poorer sexual function (26). Furthermore, racial differences in survival are enormous. Our study found that Caucasian patients were more prone to metastasis, which is consistent with previous studies (27). Relevant reports suggested that the incidence of endometrial cancer in black women was low, but black women had higher tumor invasiveness, and the mortality rate was 80% higher than that in white women (28). A study on the impact of marital status on the prognosis of patients with EC also identified marital status as conducive to patient prognoses. The risk of death of widowed and unmarried patients was higher than that of other marital statuses (29). This may be because spouses can provide social support and encourage patients to seek medical assistance. The results of this study revealed no correlation between survival and marital status in multivariate analysis. The pathological grade is an important independent factor affecting the prognosis of endometrial carcinoma. Some studies have proposed a clinical prediction model for patients with poorly differentiated endometrial cancer (30). For patients with metastatic endometrial cancer included this time, more than 30% of them were poorly differentiated, and their OS and CSS were significantly reduced. The pattern of distant metastasis appeared to be influenced by the histological type of the patient. In our study, adenocarcinoma accounted for only 30% of the cases, while other pathological types accounted for 70%. Clear cell endometrial carcinoma is purportedly more prone to bone metastasis (17). TNM staging is the current clinical staging method used to evaluate tumor surgery and prognosis. Our study shows that T staging plays an important role in distant metastasis of endometrial carcinoma. Although FIGO stage IV endometrial cancer is rare, it is associated with a high risk of early death.

This study implemented a nomogram-prediction model for endometrial cancer prognosis to provide individualized treatment for patients. For example, in a 65-year-old patient, liver metastasis of endometrial adenocarcinoma occurred at initial diagnosis. The pathological grade was grade II, T2N0M1 stage, without surgical treatment, and chemotherapy combined with radiotherapy was administered. By adding the points of each prognostic predictor, the got 342.23 and 344.88 scores in OS and CSS nomograms respectively. According to the nomogram, the 1-year OS and CSS rates were estimated to be 15% and 18%, respectively. Simultaneously, we divided all patients into high-risk and low-risk groups according to the total score of the nomogram for survival comparison. Compared with the traditional risk stratification system, the nomogram can simultaneously evaluate multiple prediction factors and accurately predict survival probability.

The strengths of our study were as follows: (I) Compared with the traditional risk stratification system, our nomogram could simultaneously evaluate multiple prediction factors and accurately predict survival probability; (II) our study included multiple metastatic sites; and (III) the inclusion of public database data in this study allows for the use of larger data and more reliable results than previous small retrospective studies. However, the current research had the following limitations: (I) a retrospective study may lead to selection bias; (II) the prediction model has not been verified externally or by other organizations; and (III) the SEER database still lacks some clinical information that is crucial to determine patient prognosis, such as lymphatic vascular invasion.

## Conclusion

In general, we discussed the prognostic factors of patients with metastatic endometrial cancer and constructed and validated a nomogram-prediction model. This model can accurately predict the survival rate of patients with metastatic endometrium and classify them according to the risk threshold of the model to better manage their prognosis.
